# Effects of Aircraft Noise Exposure on Heart Rate during Sleep in the Population Living Near Airports

**DOI:** 10.3390/ijerph16020269

**Published:** 2019-01-18

**Authors:** Ali-Mohamed Nassur, Damien Léger, Marie Lefèvre, Maxime Elbaz, Fanny Mietlicki, Philippe Nguyen, Carlos Ribeiro, Matthieu Sineau, Bernard Laumon, Anne-Sophie Evrard

**Affiliations:** 1 Université Lyon, Université Claude Bernard Lyon1, IFSTTAR, UMRESTTE, UMR T_9405, F-69675 Bron, France; marie.lef@laposte.net (M.L.); anne-sophie.evrard@ifsttar.fr (A.-S.E.); 2 Université Paris Descartes, APHP, Hôtel-Dieu de Paris, Centre du Sommeil et de la Vigilance et EA 7330 VIFASOM, 75004 Paris, France; damien.leger@aphp.fr (D.L.); maxime.elbaz@aphp.fr (M.E.); 3 Bruitparif, the Center for Technical Assessment of the Noise Environment in the Île-de-France Region of France, 93200 Saint-Denis, France; Fanny.Mietlicki@bruitparif.fr (F.M.); Philippe.NGuyen@bruitparif.fr (P.N.); Carlos.Ribeiro@bruitparif.fr (C.R.); Matthieu.Sineau@bruitparif.fr (M.S.); 4 IFSTTAR, Transport, Health and Safety Department, F-69675 Bron, France; bernard.laumon@ifsttar.fr

**Keywords:** aircraft noise, sleep, heart rate, cross-sectional study

## Abstract

*Background* Noise in the vicinity of airports is a public health problem. Many laboratory studies have shown that heart rate is altered during sleep after exposure to road or railway noise. Fewer studies have looked at the effects of exposure to aircraft noise on heart rate during sleep in populations living near airports. *Objective* The aim of this study was to investigate the relationship between the sound pressure level (SPL) of aircraft noise and heart rate during sleep in populations living near airports in France. *Methods* In total, 92 people living near the Paris-Charles de Gaulle and Toulouse-Blagnac airports participated in this study. Heart rate was recorded every 15 s during one night, using an Actiheart monitor, with simultaneous measurements of SPL of aircraft noise inside the participants’ bedrooms. Energy and event-related indicators were then estimated. Mixed linear regression models were applied, taking into account potential confounding factors, to investigate the relationship between energy indicators and heart rate during sleep measured every 15 s. Event-related analyses were also carried out in order to study the effects of an acoustic event associated with aircraft noise on heart rate during sleep. *Results* The more the SPL from all sources (L_Aeq,15s_) and the SPL exceeded for 90% of the measurement period (L_A90,15s_) increased, the more heart rate also increased. No significant associations were observed between the maximum 1-s equivalent SPL associated with aircraft overflight (L_Amax,1s_) and differences between the heart rate recorded during or 15 or 30 s after an aircraft noise event and that recorded before the event. On the other hand, a positive and significant association was found between L_Amax,1s_ and the heart rate amplitude calculated during an aircraft noise event. Results were unchanged when analyses were limited to participants who had lived more than five years in their present dwelling. *Conclusion* Our study shows that exposure to the maximum SPL linked to aircraft overflight affect the heart rate during sleep of residents near airports. However, further studies on a larger number of participants over several nights are needed to confirm these results.

## 1. Introduction

The environmental and health consequences of the increase in air traffic have led to questioning and mounting concern among residents near airports. Noise pollution around airports is the subject of constant conflict between airport authorities and local residents and is viewed as a major factor of environmental stress. According to the World Health Organisation (WHO), sleep disorder is the most serious consequence of environmental noise in Western Europe [1].

Several studies have shown a relationship between aircraft noise exposure and sleep disturbances [2]. The effects of aircraft noise on sleep can result in the modification of several physiological parameters of sleep, such as sleep onset latency, the number and duration of awakenings during sleep, the total length of sleep, modification in sleep structure (the number of changes in sleep stages and in rhythms characteristic of sleep stages), the number of body movements and of autonomic modifications during sleep such as to heart rate and blood pressure [2,3,4,5]. If noise-induced sleep disturbance is considerable and frequent, it can lead to significant fragmentation and deprivation of sleep which in turn may seriously affect one’s physical and mental health [6]. Sleep disturbances can have immediate and long-term health consequences [7].

During sleep, sympathetic tone is reduced, leading to a reduction in heart rate which becomes very regular. However, the presence of an external stimulus, such as a noise event due to aircraft overflight, is likely to accelerate the heart rate. Then, heart rate measured during sleep can be an indicator that may be used to objectively assess sleep quality [8]. Many studies have shown an increase in heart rate in persons exposed to noise from road, railway and air transportation [8,9]. Exposure to noise disturbs sleep, notably by increasing the heart rate [9], but also blood pressure [10] and hormone levels linked to stress [11]. In turn, these increases can lead to endothelial dysfunction and high blood pressure [12]. Sleep disturbances linked to exposure to transportation noise can be a factor causing cardiovascular disease [9].

Almost all studies on the effects of transportation noise on heart rate have been carried out in laboratories and looked at noise from either road or railway traffic. No similar studies have been conducted at participants’ homes, focusing on the effects of aircraft noise.

In France, a research program called DEBATS (Discussion on the Health Effects of Aircraft Noise) was set up to deepen the knowledge and understanding of the consequences of aircraft noise and to quantify its effects on the health of populations residing near French airports. One of the program’s objectives is to assess, for the first time in France, the subjective and objective sleep quality of those living near the country’s airports. DEBATS has shown that exposure to aircraft noise near airports affects the subjective quality of sleep, with an increased risk of sleeping less than 6 h and an increased feeling of tiredness in the morning [13]. Aircraft noise exposure also affected the objective parameters of sleep quality, with an increase in the time taken to fall asleep and in the total wake time after sleep onset, and a reduction in sleep efficiency. On the other hand, there was an increase in total sleep time and time spent in bed; this could be a matter of behavioral adaption to sleep deprivation [14]. The present article more specifically addresses the issue of heart rate as an objective indicator of sleep quality, and its association with aircraft noise exposure as the question of whether a rise in heart rate is linked to an increase in the level or number of aircraft noise events has been raised.

## 2. Methods

### 2.1. Study Population

The DEBATS study population comprises residents of at least 18 years of age and living in the vicinity of one of three major French airports: Paris-Charles de Gaulle, Lyon Saint-Exupéry, and Toulouse-Blagnac. In total, 1244 individuals took part in the main DEBATS study. These participants responded to a questionnaire administered by an interviewer at their home. At the beginning of the interview, they were asked if they agreed to take part in a further study aimed at better understanding the effects of aircraft noise on their sleep. Individuals who had declared that they snore during sleep or share a bedroom with a snorer were excluded. For material reasons, participants living on the ground floor of a building and overlooking a public road were also excluded. In total, 112 volunteers signed and returned their informed consent by mail and took part in the supplementary study: 91 residents near Paris-Charles de Gaulle airport and 21 residents near Toulouse-Blagnac airport. This study was limited to those two airports because contrasts in exposure to aircraft noise are sufficiently significant in their vicinity, which is not the case for Lyon Saint-Exupéry.

The present study was approved by two national authorities in France, the French Advisory Committee for Data Processing in Health Research (Project identification code: 11.405) and the French National Commission for Data Protection and the Liberties (Project identification code: 911365).

### 2.2. Aircraft Noise Exposure Assessment

Exposure to aircraft noise at home was measured continuously during eight days in order to increase the probability of measuring noise under differing meteorological conditions and activity patterns of the airport hubs. Two metrological class 1 sound level meters were installed at the home of each participant. The first was installed on the outside wall of the bedroom in line with the bedroom window and 20–25 cm in front of the façade, in order to detect acoustic events associated with aircraft noise, while the second was on the bedside table inside the bedroom in order to measure the overall interior sound pressure level (SPL). Technicians set up the equipment and collect it again at the end of measurement period. The two sound level meters were synchronized at the beginning of the measurements. However, after the measurements, the intercorrelation between both signals was calculated in order to check the temporal synchronization. If needed, the time lag was corrected. The recorded SPL correspond to L_Aeq,1s_ and the associated third octave frequency band levels. The sound level meters used complies with class 1 of the standard CEI 61672 (maximum accuracy in acoustic measurement). The processing of data associated with aircraft noise outside the dwelling is in accordance with the procedures of ISO 20906, and NF S 31-190.

Bruitparif (the center for technical assessment of the noise environment in the Île-de-France region of France, around Paris) has developed an algorithm to deduce, from these measurements, the original SPL of aircraft noise inside the bedroom. This algorithm is composed of four steps. The first step consists in determining, from the outdoor signal measured on the outer wall of the building, the acoustic events associated with aircraft overflight, based on correspondence between the radar trajectories supplied by the French Civil Aviation Authority (DGAC) and the observed acoustic events. The second step, based on the measurement taken inside the room, determined the periods affected by aircraft noise, by correlation with the times of acoustic events identified as aircraft noise in the outdoor measurement. The third step consisted of estimation of an exterior/interior transfer function, identifying acoustic signals in the room caused exclusively by the exterior noise. The final stage consisted of statistical analysis of time-matched curves for exterior and interior SPL at the dwelling, filtering out acoustic events originating indoors (e.g., snoring, clocks, or domestic animals) which occurred during overflight. This filtering is based on comparing the estimate of the sound signal inside the bedroom caused exclusively by exterior noise (using the transfer function) to the sound signal measured in the bedroom. A significant difference between the two signals corresponds to the presence of an indoor noise. Filtering in this way was necessary in order to produce reliable estimates of indicators linked to noise inside the bedroom generated by the overflight of aircraft. Energy indicators (relative to the sound energetic averages of noise for given periods) and event-related indicators (characteristics and number of events exceeding a given SPL) were derived from these measurements. They were estimated inside the participants’ bedroom, for all noise sources, but also for aircraft noise alone, every 15 s during the participant’s sleep period, which is to say between falling asleep and final awakening. Energy indicators included the A-weighted equivalent SPL from all sources (L_Aeq,15s_) and specifically from aircraft noise (L_Aeq,aero,15s_) during 15 s, and the SPL during 15 s exceeded for 90% of the measurement period (L_A90,15s_). For each acoustic event associated with aircraft noise during sleep time, two acoustic indicators were calculated inside the room: the maximum SPL observed in one second associated with an aircraft overflight (L_Amax,1s_), and the level of background noise 10 min before an aircraft overflight.

### 2.3. Heart Rate

During one of the eight nights of acoustic measurements (generally the first night, due to the device autonomy and storage capacity), the participants wore a heart rate monitor, called Actiheart. Actiheart (CamNtech, Cambridge, UK) is a compact device that records heart rate and movement. It has a sensitivity of 0.250 mV. The signal of the electrocardiogram (ECG) is sampled at 128 Hz and, at the end of each period, the adjusted average of the last 16 R-R intervals is calculated, excluding values outside ±25% of the initial average. This signal is converted into beats per minute (bpm) and stored in the device memory at the end of each period. The possible measurement span for heart rate is from 31 to 250 bpm. Explanations about the device’s functions and instructions on using it were given to participants by a Bruitparif technician during installation of the acoustic equipment. The participants were instructed to sleep under normal conditions, and to place two ECG electrodes on their upper chest before going to bed. Data were then uploaded by means of a USB reader and Actiheart software (CamNtech, Cambridge, UK) version 4.0.16, then exported to a file which enabled calculation of the heart rate. The Actiheart measurements were then used to determine the number of heart beats per minute (HR) of each participant every 15 s during their sleep. Measurements from six subjects were judged unusable because of a hardware problem. In the end, Bpm measurements <35 and >130 were excluded from analysis [9,15]: at rest, bpm below 35 or above 130 is considered abnormal [16].

The two sound level meters and the Actiheart monitor were synchronized at the beginning of the measurements to the nearest second. The recording dates of the SPL and heart rates did not match for 14 subjects who were therefore excluded from analysis. In the end, measurements of 92 of the 112 subjects were used in the analyses for a total of 92 nights.

### 2.4. Statistical Analysis

#### 2.4.1. Energy Indicators

In total, 141,595 observations were used to estimate the models: indeed, for each participant, a little more than 1500 L_Aeq,15s_ have been linked to as many HR measured every 15 s.

Mixed univariate (Model 1) and multivariate (Model 2) linear regression models were used to study the association between the SPL of aircraft noise characterized by energy indicators (L_Aeq,15s_, L_Aeq,aero,15s_ and L_A90,15s_) and heart rate during sleep measured every 15 s. The multivariate models (Model 2) were adjusted on risk factors known in the literature to have an influence on heart rate: gender, age, body mass index (BMI), physical exercise, smoking and alcohol consumption [17,18]. As the presence of cardiovascular or hypertensive problems could modify the association between aircraft noise exposure and heart rate during sleep, they were also included in the models (Model 2). These factors were either collected through the questionnaire used in the interview with participants, or through objective assessments made by the interviewers. The time elapsed since the onset of sleep, determined through measurements with a wrist actigraph, was also introduced into the multivariate models [14,19].

#### 2.4.2. Event-Related Indicators

To study changes to heart rate during sleep that are linked to an acoustic event associated with aircraft noise, the heart rate measured 15 s before an event was considered as baseline. This was compared to the mean heart rate during the event, and then 15 s and 30 s after the event. Three variables were thus constructed:

- HR1 = the difference between the heart rate during the event and the heart rate before the event in beats per minute,
- HR2 = the difference between the heart rate 15 s after the event and heart rate before the event in beats per minute,
- HR3 = the difference between the heart rate 30 s after the event and the heart rate before the event in beats per minute.

Finally, another variable, HRA-, was determined: heart rate amplitude during an acoustic event implicating aircraft noise. HRA was calculated as the difference between the maximum and minimum heart rate during an acoustic event, in beats per minute.

No aircraft-related event was detected overnight for six participants. Thus, the event-related analyses included 86 subjects and used 3081 observations corresponding to the total number of events related to aircraft noise to estimate the coefficients.

Mixed linear models including a random intercept were applied in order to assess the effects of aircraft noise events characterized by the L_Amax,1s_ on heart rate during sleep defined by the variables HR1, HR2, HR3 and HRA. In addition to the previously cited risk factors known in the literature to influence heart rate, and the time elapsed since the onset of sleep, the multivariate models were also adjusted on the background SPL 10 min before the onset of the event. The interaction between the background SPL 10 min before the onset of the event and L_Amax,1s_ was included initially in the multivariate regression model, but did not contribute significantly to the model and had no impact on estimating the noise effect, and was therefore not included in the final model.

Sensitivity analyses were carried out on the population sample who had resided longer than 5 years in their present dwelling.

Analyses were performed using SAS software version 9.4 (SAS Institute Inc., Cary, NC, USA).

### 2.5. Ethics Approval

Two national authorities in France, the French Advisory Committee for Data Processing in Health Research and the French National Commission for Data Protection and the Liberties approved the present study.

## 3. Results

Table 1 describes the sociodemographic characteristics of the 92 participants for whom the acoustic and Actiheart measurements were available. Their sociodemographic characteristics were similar to those of participants in the main DEBATS study, although the proportions of females, young adults aged between 18 and 34, and singles were higher in our study (61%, 26% and 32% respectively) than in the main DEBATS study (56%, 18% and 20% respectively). The proportion of obese persons was conversely lower in our study (10% against 20% in the main study).

**Figure 1:**
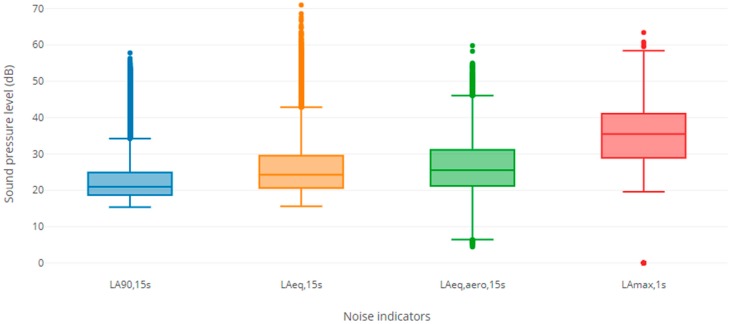
Box plots of the sound level indicators measured indoors during the Actiheart measurement night.

The average level of 15-s equivalent SPL inside the bedroom was 26 dB(A) for all sources of noise taken together (L_Aeq,15s_) and 27 dB(A) for aircraft noise alone (L_Aeq,aero,15s_). The average 15-s equivalent SPL exceeded for 90% of the measurement period (L_A90,15s_) inside the bedroom was 23 dB(A) (Figure 1).

Table 2 reports the average number of events linked to aircraft noise at night and their duration. On average, 30 acoustic events linked to aircraft noise were detected and recorded in the course of one night, with an average maximum SPL associated with overflight of an aircraft (mean L_Amax,1s_) of 31 dB(A). The average duration of an acoustic event linked to aircraft noise was 1 min 41 s. Table 2 also presents the heart rate characteristics (HR) of each participant, and the heart rate variables used in the models (HR1, HR2, HR3 and HRA). For the 92 participants as a whole, the average heart rate during sleep was 65 bpm. The differences between the heart rate during or 15 or 30 s after the event and before the event (HR1, HR2 and HR3) were very minor. The average amplitude of the heart rate during an acoustic event associated with aircraft was 6.21.

### 3.1. Energy Indicators

Positive and significant associations were highlighted between the energy indicators (L_Aeq,15s_ and L_A90,15s_) and the heart rate (HR) measured every 15 s during sleep (Table 3). An increase in the SPL inside the bedroom, from all sources of noise, or exclusively linked to aircraft noise, or exceeded for 90% of the measurement period, significantly increased heart rate during sleep. For example, a 10 dB(A) increase in L_Aeq,15s_ was associated with an increase of 0.71 bpm in HR. This increase was greater for the SPL from all sources (L_Aeq,15s_) than for the SPL exceeded for 90% of the measurement period (L_A90,15s_) or for the SPL due to aircraft (L_Aeq,aero,15s_). The association between the SPL exclusively linked to aircraft noise (L_Aeq,aero,15s_) and heart rate was not significant in the multivariate models.

### 3.2. Event-Related Indicators

Table 4 presents the results from models assessing the effects of acoustic events linked to aircraft noise on the heart rate during sleep, characterized by the variables HR1, HR2, HR3 and HRA. Whatever the model, no significant association was found between aircraft noise exposure characterized by the event indicator L_Amax,1s_ and the differences between the heart rates recorded during or 15 or 30 s (HR1, HR2 and HR3) after the aircraft noise event and the heart rate before the event. In contrast, the univariate and multivariate models highlighted a significant positive association between L_Amax,1s_ and the heart rate amplitude during an aircraft noise event (HRA).

Limiting the analyses to participants who had occupied their present dwelling for at least 5 years did not affect the results.

## 4. Discussion

The present study showed a significant increase in heart rate during sleep with increasing equivalent SPL inside the bedroom of all noise sources (L_Aeq,15s_), and with increasing SPL exceeded for 90% of the measurement period inside the bedroom (L_A90,15s_). This result confirms the findings obtained in the literature [8]. In the field, Wilkinson and Campbell also reported that an increase in road traffic noise (L_Aeq_) showed a significant positive correlation with elevated heart rate [20]. Griefahn and Gros also found, in the field, a significant positive correlation between the equivalent level of railway noise calculated for each minute (L_Aeq,1min_) and heart rate [21]. In the laboratory, Tassi et al. showed a dose-effect relationship between the level of railway noise and heart rate during sleep [22].

On the other hand, contrary to almost all studies that found an increase in heart rate after an acoustic event linked to transportation noise [9,23,24], this study did not show a significant increase in heart rate linked to the maximum SPL associated with aircraft overflight (L_Amax,1s_). Griefahn et al. highlighted an increase in heart rate after a noise event related to road, railway or air transportation at maximum levels (L_Amax_) from 45 to 77 dB(A) [9]. Öhrström et al. showed a significant 1.8 bpm increase in heart rate after a noise event linked to road traffic for individuals sensitive to noise and a 1.1 bpm increase for individuals non-sensitive to noise [23]. In another experiment, while the authors did not find an association between average heart rate over total sleep duration and exposure to road traffic noise, they did observe a significant increase in heart rate immediately following an acoustic event linked to road traffic [24].

Acoustic events associated with aircraft noise detected in our study were found to have low levels of L_Amax,1s_ inside the bedroom (mean: 31 dB(A)). These low levels of L_Amax,1s_ could explain the fact that no significant increase in heart rate after the passage of an aircraft (HR1, HR2, HR3) was observed in the present study. In most studies investigating the effects of transportation noise on heart rate during sleep, noise levels were higher, rarely lower than 45 dB(A) [9,23,24,25,26]. In the study by Griefahn et al., the average levels of L_Amax_ were 55 dB(A) for aircraft noise events [9], while in the one by Öhrström et al., the subjects were exposed to at least 50 dB(A) in terms of L_Amax_ [23].

As in the majority of studies, the present study showed that the amplitude of heart rate during an aircraft noise event increased significantly with the maximum level of aircraft noise (L_Amax,1s_). Tassi et al. found a dose-response relationship between the maximum level associated with a railway noise event (L_Amax_) and the amplitude of the cardiac response during the event [22]. In another study, the maximum level of night-time noise from trains (L_Amax_) also led to a large significant increase in the amplitude of cardiac response during the event [27]. Di Nisi et al. showed that the amplitude of heart rate during the event was higher during air and railway noise events than during events linked to the noise of trucks and motorbikes [28].

In the present study, the results of sensitivity analyses limited to participants living more than five years at their current dwelling were similar to those obtained for the whole population. Therefore, there does not seem to exist an habituation of heart rate to aircraft noise events in our population, which confirms the results of the majority of studies [8,9,23]. Öhrström et al. found that the cardiac reaction to exposure to road traffic noise was as pronounced at the end as at the beginning of the noise exposure period [23]. Griefahn et al. observed that cardiac responses did not diminish in the course of the night [9].

The first strength of this study concerns the measurement by an Actiheart monitor of participants’ heart rates while asleep at home as an objective indicator of sleep quality. The participants had to sleep in their usual conditions. Most studies of the effects of transportation noise in general, and aircraft noise in particular, on heart rate during sleep were carried out in a laboratory and used an electrocardiogram (ECG) to measure heart rate. Only the study by Haralabidis et al. was conducted in the field [27]. That study did not find a significant association between the maximum level of noise associated with overflight of an aircraft (L_Amax_) and heart rate measured by means of a “Mobilograph” device [27]. Using an ECG allowed these studies to estimate heart rate beat-by-beat. This showed an immediate increase in the number of heart beats following the onset of the noise event. Griefahn et al. reported that this increase reached a maximum 13.2 s after the onset of the event and was immediately followed by a deceleration in the number of heart beats [9,29]; the heart rate became stable between 30 and 40 s after the onset of the event [9]. Our study was the first to use an Actiheart monitor to measure heart rate during sleep, and this device does not allow a beat-by-beat study. It is difficult and costly to use an ECG at a subject’s home and with large-scale samples. The Actiheart monitor is a small light-weight device that can be easily worn on the chest and does not require the attendance of a medical professional. It is commonly used for monitoring heart rate during physical exercise. One study showed that heart rate measured by an Actiheart monitor was similar to that measured by ECG [29]. Our results are compatible with the hypothesis that cardiac response increases immediately after onset of a noise event but stabilizes before or less than 15 s after the end of the event.

The second strength of this study concerns acoustic measurements taken at participants’ homes from which the energy- and event-related indicators were estimated inside the bedroom. In the field, the majority of studies did not have control over levels of noise exposure, unlike laboratory studies [30]. At the homes of some participants, several noise events linked to airplanes followed one another at intervals of less than a second during the night, a situation quite different from laboratory studies, where noise events are generally more spaced out. Furthermore, the acoustic measurements performed during this study allowed us to estimate not only the energy indicators but also event-related indicators. The energy indicators are recommended by the European Directive of 2002 [31] relating to the assessment and management of environmental noise. At the present time, however, event-related indicators are preferred for studies of the effects of noise on sleep, as they can better characterize aircraft noise [32]. The WHO also pointed out that event-related indicators seemed to be more predictive of sudden short-term effects, such as movement onset, awakening, cardiovascular response and change in sleep stage [1]. Our study, like most of the literature, showed an increase in heart rate with exposure to aircraft noise, whether characterized by energy indicators or event-related indicators.

Nevertheless, a selection bias cannot be excluded from our study. Participants were recruited on a voluntary basis from the main DEBATS study, and therefore may have been more concerned about noise pollution and more annoyed by it. Indeed, 43% of participants in this Actiheart study reported sleep problems, compared to 32% in the main DEBATS study. In the present study, 32% of subjects stated they felt tired on waking up in the morning, against 30% in the main study and 24% of participants in the Actiheart study stated that they felt extremely or greatly bothered by aircraft noise, compared to 18% in the main DEBATS study. Thus, mild selection bias probably cannot be excluded in interpreting the results of the present study.

One of the limitations of our study concerns the fact that we lacked information on possible awakening when an aircraft noise event occurred during the sleep period. Heart rate responses to transportation noise can differ according to whether the subject has been awakened or not by the noise event [9]. If the subject is awakened, monophasic elevations lasting more than one minute are observed, with an average maximum elevation of 30 beats per minute. If the subject is not awakened, the heart rate responses are biphasic with accelerations (maximal elevation of 9 bpm) followed by decelerations below baseline [9]. In our study, the subjects also wore a wrist actigraph during the night [14]. The actigraph recorded the rest/activity rhythm which enabled assessment of the wake/sleep rhythm based on accelerations linked to movements; a total of 20 movements per minute is sufficient to designate a period as an “awake” state [30,33]. However, the present study could not take this information into account, because a relatively weighty supplementary processing of raw data would have been needed to obtain this information every 15 s; this will be undertaken shortly. Furthermore, heart rate does not remain the same throughout the various stages of sleep. Tassi et al. showed that noise from trains at night had significant effects on heart rate that were greater during stage 2 (light slow-wave sleep) and REM sleep (stage 4) than in deep slow-wave sleep (stage 3) [27]. However, the present study did not determine the internal sleep structure of the participants and was consequently unable to study the effects of aircraft noise on heart rate according to sleep stage. It would be very difficult and costly to carry out polysomnographic measurement at the homes of participants in order to determine the internal structure of their sleep.

One other limit of this study is that heart rate was assessed during one single night and it may be possible that this night was not representative of the ordinary nights of the participants. However, it has been assumed that placing two electrodes on the subjects’ thorax would not significantly change the sleep of the subjects, as is usually the case when recording polysomnography in sleep laboratories, which is called the “first night effect”. The main strength of this study is that it was conducted at the participants’ homes, whereas all studies on the subject were conducted in laboratories. Participants did not need to adhere to specific sleep times during the study period. They should behave as much as possible as usual in order to collect parameters that were representative of everyday life. Moreover, in the present study, we did not assess sleep but the impact of noise on heart rate during sleep. In addition, the autonomy and storage capacity of the Actiheart monitor did not allow us to record heart rate over several nights.

## 5. Conclusions

This study is one of the few in Europe to assess the association between the sound pressure level (SPL) of aircraft noise on heart rate during sleep at the dwellings of residents near airports. It showed that heart rate increased with increasing equivalent SPL of noise from all sources (L_Aeq,15s_) and increasing SPL of noise exceeded for 90% of the measurement period (L_A90,15s_). Our study found a significant positive association between the level of maximum noise associated with an aircraft overflight (L_Amax,1s_) and the amplitude of the heart rate during this aircraft noise event. However, further studies carried out on a larger number of subjects and over several nights would be needed to confirm all of these results.

## Figures and Tables

**Table 1:** Description of the 92 participants in the Actiheart study and of the 1244 participants in the main DEBATS (Discussion on the Health Effects of Aircraft Noise) study.

Characteristics of the Participants	Actiheart Study *n* = 92		Main Study *n* = 1244	
	*n*	(%)	*n*	(%)
Gender				
Female	56	(61)	695	(56)
Male	36	(39)	549	(44)
Age (years)				
18–34	24	(26)	226	(18)
35–44	17	(18)	236	(19)
45–54	23	(25)	266	(21)
55–64	13	(14)	260	(21)
65–74	8	(9)	185	(15)
75+	7	(8)	71	(6)
Marital status				
Single	29	(32)	253	(20)
Married	46	(50)	782	(63)
Widowed	3	(3)	76	(6)
Divorced	14	(15)	133	(11)
Education				
<French high school certificate	33	(36)	452	(36)
French high school certificate	27	(29)	397	(32)
>French high school certificate	32	(35)	395	(32)
BMI				
Underweight or normal weight	51	(55)	562	(46)
Overweight	32	(35)	424	(34)
Obese	9	(10)	249	(20)
Smoking habits				
Non smoker	52	(57)	625	(50)
Ex-smoker	19	(21)	330	(27)
Occasional and Daily smoker	21	(23)	288	(23)
Alcohol consumption				
No	35	(38)	348	(28)
Light	46	(50)	638	(52)
Moderate and Heavy	11	(12)	247	(20)
Physical activity				
No	46	(50)	587	(47)
Low	26	(28)	366	(29)
Moderate	15	(16)	182	(15)
Intense	5	(5)	106	(9)
Cardiovascular disease				
No	83	(90)	1088	(87)
Yes	9	(10)	156	(13)
Hypertension				
No	64	(70)	804	(65)
Yes	28	(30)	426	(35)

**Table 2:** Description of the outcomes and sound level indicators.

Outcomes and Acoustic Indicators	Mean	SD	P5	P95
Outcomes				
HR	65	8	51	80
HR1	0.32	1.59	−1.14	2.00
HR2	0.01	1.21	−2.03	1.71
HR3	−0.06	1.92	−2.15	2.00
HRA	6.21	2.96	2.17	12.06
Events indicators				
Number of aircraft events per night	30	23	2	72
Event duration (h:m:s)	00:01:41	00:00:33	00:00:46	00:02:37
L_Amax,1s_ (dB(A))	31	16	0	50
Energy indicators				
LAeq,15s	26	7	18	40
L_Aeq,aero,15s_	27	7	16	40
L_A90,15s_	23	6	17	33

HR1 = heart rate during event − heart rate before event; HR2 = heart rate 15 s after event − heart rate before event; HR3 = heart rate 30 s after event − heart rate before event; HRA = Amplitude during event.

**Table 3:** Association between energy indicators estimated every 15 s and heart rate measured every 15 s during the sleep period (HR).

Energy Indicators	Model 1	Model 2
	Estimate (95% CI)	Estimate (95% CI)
L_Aeq,15s_ (dB(A)) *	**0.85 (0.79;0.90)**	**0.71 (0.65;0.76)**
L_Aeq,aero,15s_ (dB(A)) *	**0.17 (0.04;0.30)**	0.005 (−0.13;0.14)
L_A90,15s_ (dB(A)) *	**0.44 (0.36;0.52)**	**0.18 (0.10;0.25)**

Model 1: univariate model including energy indicators separately; Model 2: multivariate model including energy indicators separately together with gender, age, body mass index (BMI), physical activity, tobacco consumption, alcohol consumption, cardiovascular disease, hypertension and elapsed time after sleep onset. * Per 10 dBA increase; Bold values are statistically significant *p* < 0.05.

**Table 4:** Analysis of event-related heart rate response.

L_Amax,1s_	HR1	HR2	HR3	HRA
	Estimate (95% CI)	Estimate (95% CI)	Estimate (95% CI)	Estimate (95% CI)
Model 1	−0.01 (−0.11;0.09)	0.03 (−0.11;0.17)	0.02 (−0.11;0.16)	**0.27 (0.06;0.47)**
Model 2	−0.04 (−0.15;0.07)	−0.02 (−0.18;0.14)	−0.04 (−0.19;0.11)	**0.34 (0.13;0.55)**

Model 1: univariate model including L_Amax,1s_; Model 2: multivariate model including L_Amax,1s_ gender, age, body mass index (BMI), physical activity, tobacco consumption, alcohol consumption, cardiovascular disease, hypertension, elapsed time after sleep onset, and background 10 min before event; Per 10 dBA increase; HR1 = heart rate during event—heart rate before event; HR2 = heart rate 15 s after event—heart rate before event; HR3 = heart rate 30 s after event—heart rate before event HRA = Amplitude during event; Bold values are statistically significant *p* < 0.05.
